# Left ventricular mass estimation by different partition values in a large population of black hypertensive subjects

**DOI:** 10.1002/hsr2.25

**Published:** 2018-01-25

**Authors:** Dike B. Ojji, Elena Libhaber, Jacob Alfa, Ngabea Murtala, Bolaji Abdullahi, Ada Nwankwo, Anita Nnamonu, Sliwa Karen

**Affiliations:** ^1^ From Cardiology Unit, Department of Medicine University of Abuja Teaching Hospital Abuja Nigeria; ^2^ South Africa Cardiovascular Research Unit, School of Clinical Medicine University of Witwatersrand South Africa; ^3^ Hatter Institute for Cardiovascular Research in Africa, Department of Medicine, Faculty of Health Sciences University of Cape Town South Africa

**Keywords:** left ventricular, mass, estimation, partition, hypertensives, Blacks

## Abstract

**Aims:**

Our aim is to compare the impact of the 2 most widely used methods of indexing left ventricular mass (LVM) on the distribution of abnormal left ventricular (LV) geometric patterns, in a large sample of untreated asymptomatic black hypertensive subjects.

**Methods and Results:**

All patients with hypertension referred to the Cardiology unit of University of Abuja Teaching Hospital, Abuja, Nigeria from 2006 to 2013, who gave informed consent, and underwent physical examination and echocardiography. LVM indexation was classified into 4 geometric patterns after echocardiography: normal geometry, concentric hypertrophy, concentric remodeling, and eccentric hypertrophy. Concentric hypertrophy was the commonest geometric pattern and was detected in 33.6% to 39.5% of the patients. LVM/height^2.7^ was a better method to detect abnormal geometric pattern than LVM/BSA (P < 0.0001).

**Conclusion:**

In a large cohort of hypertensive subjects with no clinical evidence of cardiovascular disease, abnormal LV geometry was found in greater than four‐fifths of the population. In addition, LVM indexed for height ^2.7^ was found to be a better method for detecting LVH than LVM indexed for BSA, as the highest prevalence of abnormal geometry was diagnosed when LVM was indexed for height^2.7^.

## Abstract 2

The 2 most widely used methods of indexing left ventricular mass (LVM) on the distribution of abnormal left ventricular (LV) geometric patterns were applied in a large population of untreated asymptomatic black hypertensive subjects with no clinical evidence of coronary artery disease. Abnormal LV geometry was found in greater than 80% of the population, concentric hypertrophy was the commonest geometric pattern, and LVM indexed for height^2.7^ was a better method than LVM indexed for BSA in detecting LVH.

## 1 INTRODUCTION

Left ventricular hypertrophy (LVH), as assessed by echocardiography, has been shown to be a strong and independent predictor of adverse prognosis in cardiovascular disease.^1^, ^2^, ^3^ LVH is often further classified according to geometric pattern into 4 types: concentric hypertrophy, eccentric hypertrophy, concentric remodeling, and normal geometry.^4^ This classification is important, as several studies have shown that subjects with concentric hypertrophy have the highest risk of cardiovascular events and deaths compared with those with concentric remodeling. Those with eccentric hypertrophy have intermediate risk, while those subjects with normal LV geometric pattern have the lowest risk of cardiovascular events.^5^, ^6^, ^7^, ^8^ The study by de Simone et al,^9^ using the Dallas Heart Study classification, has further described the mechanism of geometric adaptation in hypertension, by showing that at any given normal ejection fraction, the balance between volume load co‐existing and pressure overload of hypertension influences the shape of LV geometric adaptation and the amount of left ventricular mass (LVM) and can impact prognosis. Therefore, the prognostic impact of LV geometry does not only depend on LVM but also on volume overload.

Different partition values have been used in different studies to characterize LV geometric patterns.^5^, ^6^, ^7^, ^8^ However, no large‐scale study has evaluated the effect of different indexation methods and partition values for LVH in hypertensive Black Africans. Other studies on LVM indexation in this population group used only LVM indexed for BSA, with no comparison to other methods of indexation.^10^, ^11^, ^12^, ^13^ The present study compares the 2 most widely used methods in cardiovascular medicine for indexing LVM^6^, ^7^, ^14^ on the distribution of abnormal LV geometric patterns, in a large sample of untreated asymptomatic Black hypertensive subjects.

## 2 METHODS

This is a prospective cohort study of new hypertensive out‐patient referrals from both Family and General Physicians (from April 2006 to August 2014) to the Cardiology Clinic of University of Abuja Teaching Hospital, Gwagwalada, Abuja, Nigeria. Out of 2001 subjects who were initially recruited for the study, 1834 were enrolled. The 167 patients excluded were those with a clinical history of angina, ECG features of myocardial infarction and/or elevated cardiac troponin I (>0.5 ng/mL), those with heart failure, stroke, diabetes, chronic kidney disease, or serum creatinine greater than 2 mg/dL, and those with regional wall motion abnormalities on the transthoracic echocardiogram. The diagnosis of hypertension was made according to the seventh guidelines of the Joint National Committee.^15^ Detailed clinical data were obtained using case report forms given to the subjects on entry. The study was carried according to the guidelines and declaration of Helsinki, and all participants provided written informed consent. The study was approved by the University of Abuja Teaching Hospital Ethics Committee. All patients had fasting blood sugar, fasting lipid profile, electrolytes, urea and creatinine, and full blood count assessed.

### 2.1 Transthoracic echocardiography

All patients underwent echocardiography using the commercially available echocardiography machine Vivid E (CE0197), Rev 4, 2010. Echocardiography examination was carried out in the left lateral decubitus position using standard parasternal, short‐axis, and apical views. All studies were carried out by trained cardiologists based on the recommendations of the American Society of Echocardiography.^16^

Measurements taken were averaged over 3 cardiac cycles, and the LV measurements taken include inter ventricular septal thickness at end diastole (IVSTd), posterior wall thickness at end diastole (PWTd), the left ventricular internal diameter in diastole (LVIDD), and left ventricular internal diameter in systole (LVIDS). Teichholz's formula^17^ was used to calculate LV systolic function. Left ventricular mass (LVM) was calculated using the formula: LVM = 0.8 [1.04 (IVSTd + LVIDD + PWTd) ^3^ − (LVIDd)^3^] + 0.6g. This formula yields values closely related to necropsy LV weight with excellent inter‐study reproducibility (*r* = 0.90).^18^The formula 2 × PWTd / LVIDD was used to calculate relative wall thickness, and increased RWT was considered present when this ratio exceeded 0.43.^18^

For LVM/body surface area (BSA), we used a cut‐off value of 116 g/m^2^ for men and 104 g/m^2^ for women. For LVM/HT^2.7^, we used a cut‐off value of 49.2 gm/m^2.7^ for men and 46.7 gm/m^2.7^ for women.^4^ Normal geometry was considered present when there was both normal left ventricular mass index (LVMI) and relative wall thickness, whereas concentric remodeling was identified by normal LVM but increased RWT. Eccentric hypertrophy, on the other hand, was said to be present when there was increased LVMI but normal RWT, while concentric hypertrophy was identified by increased LVMI and RWT.

### 2.2 Statistical analysis

Data analysis was carried out using SPSS version 16.0 software (SPSS, Chicago IL, USA). Continuous variables are expressed as mean ± SD, while categorical variables are expressed as percentages. To assess the normality of continuous variables, the Kolmogorov‐Smirnov statistics was used. One‐way ANOVA with Sheffe's post hoc test was used to assess comparison between the groups, while chi‐square was used to compare proportions. A 2‐tailed *P*‐value ≤ 0.05 was considered statistically significant.

## 3 RESULTS

### 3.1 Clinical and demographic data of the subjects according to the two partition values

Table 1 shows that independent of whether LV mass index was estimated using the LVM/BSA or LVM/HT^2.7^ indexation method, patients with concentric remodeling were the oldest.

**Table 1:** Baseline characteristics of the 3 left ventricular geometric patterns using left ventricular mass indexed for body surface area

Variable	Concentric hypertrophy (33.6%)	Eccentric hypertrophy (30.6%)	Concentric remodeling (19.8%)	Normal geometry (16%)	*P*‐Value
Age, years	52.6 ± 12.2	50.5 ± 12.8	53.9 ± 12.5^b^	51.6 ± 11.8	<0.001
BMI, kg/m^2^	29.5 ± 6.3	29.7 ± 6.6^a^	28.7 ± 6.4	28.3 ± 6.0	0.017
PP, mmHg	52.6 ± 16.4	51.8 ± 16.6	51.2 ± 16.2	52.0 ± 16.1	0.57
MAP, mmHg	116.8 ± 18.2	112.6 ± 18.6	114.7 ± 17.3	115.5 ± 17.4	0.55
FPG, mmol/L	5.3 ± 0.6	5.5 ± 0.5	5.4 ± 0.4	5.3 ± 07	0.41
Serum creatinine, mmol/L	94.1 ± 9.3	96.2 ± 8.6	93.4 ± 8.6	93.5 ± 8.1	0.31
Total cholesterol, mmol/L	4.4 ± 1.1	4.3 ± 1.2	4.5 ± 1.0	4.2 ± 0.9	0.20
LDL cholesterol, mmol/L	3.7 ± 0.8	3.5 ± 0.5	3.6 ± 0.6	3.7 ± 0.5	0.42
HDL cholesterol, mmol/L	1.4 ± 0.2	1.4 ± 0.4	1.4 ± 0.3	1.3 ± 0.5	0.68
Packed cell volume, %	38.1 ± 6.6	37.5 ± 6.6	37.9 ± 6.4	38.6 ± 6.3	0.12

Abbreviations: BMI, body mass index; FPG, fasting plasma glucose; HDL, high density lipoprotein; LDL, low density lipoprotein; MAP, mean arterial pressure; PP, pulse pressure.

a Significantly higher compared with normal geometry (*P* = 0.017).

b Significantly higher compared with eccentric hypertrophy (*P* < 0.01).

Table 2 shows that the largest BMI was found in patients with eccentric hypertrophy, independent of the method of indexation used. By using LVM/HT^2.7^, we found that patients with eccentric hypertrophy had BMIs that were significantly higher than those in patients with any of the other three geometric patterns. When the LVM/BSA method was used, however, we observed that patients with eccentric hypertrophy had BMIs that were only higher than those in patients with normal geometry.

**Table 2:** Baseline characteristics of the 3 left ventricular geometric patterns using left ventricular mass indexed for height^2.7^

Variable	Concentric hypertrophy (39.5%)	Eccentric hypertrophy (33.2%)	Concentric remodeling (17.4%)	Normal geometry (10.0%)	*P*‐Value
Age, years	53.4 ± 10.3^b^	50.4 ± 11.8^a^	53.9 ± 11.5^c^	45.5 ± 10.5	<0.001
BMI, kg/m^2^	27.8 ± 6.2	29.7 ± 6.2^d^	28.7 ± 6.4	27.1 ± 6.1	<0.001
PP, mmHg	51.7 ± 15.5	51.8 ± 16.8	50.9 ± 16.2	50.2 ± 16.1	0.57
MAP, mmHg	113.8 ± 18.2	116.6 ± 18.6	114.7 ± 17.3	115.6 ± 17.6	0.54
FPG, mmol/L	5.3 ± 0.6	5.5 ± 0.5	5.4 ± 0.4	5.3 ± 0.3	0.42
Serum creatinine, μmol/L	96.1 ± 9.5	97.2 ± 8.8	95.4 ± 8.7	96.5 ± 8.6	0.32
Total cholesterol, mmol/L	4.6 ± 1.1	4.4 ± 1.3	4.7 ± 0.5	4.5 ± 1.2	0.22
LDL cholesterol, mmol/L	3.5 ± 0.7	3.6 ± 0.4	3.7 ± 0.6	3.8 ± 0.5	0.44
HDL cholesterol, mmol/L	1.4 ± 0.2	1.4 ± 0.4	1.4 ± 0.3	1.4 ± 0.5	0.68
Packed cell volume, %	38.2 ± 6.6	38.5 ± 6.6	37.9 ± 6.4	38.4 ± 6.5	0.11

BMI, body mass index; FPG, fasting plasma glucose; HDL, high density lipoprotein; LDL, low density lipoprotein; MAP, mean arterial pressure; PP, pulse pressure.

a Significantly higher compared with normal geometry (*P* < 0.001).

b Significantly higher compared with eccentric hypertrophy and normal geometry.

c Significantly higher compared with eccentric hypertrophy and normal geometry (*P* < 0.001).

d Significantly higher compared with concentric hypertrophy, concentric remodeling, and normal geometry (*P* < 0.001).

### 3.2 Distribution of LV geometric patterns by percentage in men and women with different modes of LVMI and partition values

Table 3 shows that female subjects had significantly worse geometric pattern when LVM was indexed for BSA, but there was no difference when indexed for height^2.7^.

**Table 3:** Distribution of LV geometric patterns by percentage in men and women with different modes of left ventricular mass index and partition values

Variable	Men/Women	NG %	CR %	EH %	CH %	AG %	*P*‐Value
LVM/BSA	116/104	18.0/14.0	17.1/22.3	29.3/31.8	35.6/31.8	82.0/86.0	0.02
LVM/HT^2.7^	49.2/46.7	10.3/9.4	18.1/16.7	31.0/35.3	40.6/38.6	89.7/90.5	0.62

AG, abnormal geometry; BSA, body surface area; CH, concentric hypertrophy; CR, concentric remodeling; EH, eccentric hypertrophy; LVM, left ventricular mass; NG, normal geometry.

Male patients were found to exhibit more concentric hypertrophy than female patients when LVM was indexed for BSA and height^2.7^. Female patients, on the other hand, exhibited more eccentric hypertrophy than male patients, irrespective of the LVM index method used. Further, male subjects exhibited more concentric remodeling than female patients when LVM was indexed for height ^2.7^ compared with when it was indexed for LVM/BSA.

## 4 DISCUSSION

This study in a large cohort of Black hypertensive subjects has revealed a range of abnormal left ventricular geometric pattern from 82% to 90%, depending on the LVM indexation method used. The prevalence of abnormal left ventricular (LV) geometry is higher than that reported in the LIFE study,^19^and by Cuspidi and co‐workers.^20^ This is not surprising, as the target populations are different. Whereas the population in the LIFE study and the study by Cuspidi and co‐workers were mainly Caucasians, our study was in Black subjects. It is well known that Black hypertensive subjects have higher prevalence of LVH compared with Caucasians.^21^ This is also supported by the findings of the Echo Normal study, in which racial differences in the prevalence of LVH were found, in spite of the use of the same cut‐off values in defining LVH.^22^ Concentric hypertrophy was found to be the most common geometric pattern in this cohort, ranging from 31.8% to 40.6%, similar to the finding of Libhaber and co‐workers,^23^ who by using the indexation method of LVM indexed for height^2.7^, found concentric hypertrophy to account for approximately 51% of the abnormal geometric pattern in their cohort. We had also earlier demonstrated concentric hypertrophy to be the most common geometric pattern in a small cohort of our hypertensive subjects.^24^ The Life study, 19 unlike the present study, showed eccentric hypertrophy as the most common form of abnormal geometry. This can be accounted for by the differences in the 2 populations studied. Apart from the difference in race/ethnicity, subjects in the LIFE study were patients with evidence of target organ damage and presence of clinical cardiovascular disease, while our subjects had no clinically established cardiovascular disease.

Although concentric hypertrophy was the commonest form of abnormal geometry in our cohort, this was based on the 2‐tiered classification of LVH, which has some limitations. Therefore, application of the 4‐tiered classification of the Dallas Heart study,^25^ which sub‐classifies concentric and eccentric hypertrophy yielding 4 distinct geometric patterns, as a follow‐up to this study, may be a better method of assessing the prevalence of concentric hypertrophy in this cohort.

The highest prevalence of abnormal geometry was diagnosed when LVM was indexed for height^2.7^. The finding that LVM indexed for height^2.7^ is a better method for detecting abnormal LV geometry is a very important one, as this indexation method has been reported by De Simone and co‐workers to offer the most accurate estimation of LVH and risk factor for pathologic changes in heart structure.^26^ Zoccali and co‐workers also found LVM indexed for height^2.7^ to be a better predictor of cardiovascular events compared with LVM indexed for BSA, in a group of patients undergoing haemodialysis.^27^ In addition, LVM to algometric signal of height^2.7^ has now been acknowledged by guidelines as the best indexation method in hypertension.^28^

Other workers have, however, found that LVM indexed for height^1.7^ is better than LVM indexed for height^2.7^ for normalizing LVM for body size.^29^

One other notable result of this study is the fact that LVM indexed for height^2.7^ gave a more similar distribution geometric pattern in men and women, while LVM/BSA resulted in unequal distribution of geometric pattern between men and women. This further strengthens the fact that LVM indexed for height^2.7^ might be a better indexation method in hypertensive patients compared with LVM indexed for BSA, as previously reported.^28^

In addition, we found that irrespective of whether LVM/BSA or LVM/HT^2.7^ was used as the method of LVM indexation, patients with concentric remodeling were the oldest, while patients with eccentric hypertrophy had the largest body mass index. Similar findings have been reported in some cohorts in sub Saharan Africa, showing the effect of age and weight on left ventricular geometry in hypertensive subjects.^30^, ^31^, ^32^

Finally, abnormal geometry was more common in female patients using all 3 indexation methods. This is similar to the findings in the LIFE Study,^14^ and those by Adebiyi and co‐workers.^10^ The most plausible reason for this is the fact that men have larger BSA and are taller than their female counterparts, on average. Another possible reason for this, as proposed by De Simone et al, is the effect of biological factors especially associated with female fat deposition.^33^

## 5 CONCLUSION

In a large cohort of hypertensive subjects with no clinical evidence of cardiovascular disease, abnormal left ventricular geometry was found in greater than four‐fifths of the population. In addition, LVM indexed for height ^2.7^was found to be a better method than LVM indexed for BSA in diagnosing LVH.

## AUTHOR CONTRIBUTIONS

Conceptualization: Dike Ojji

Data Curation: Dike Ojji, Jacob Alfa, Ngabea Murtala, Bolaji Abdullahi, Ada Nwankwo, Anita Nnamonu

Formal Analysis: Dike Ojji, Elena Libhaber

Investigation: Dike Ojji, Jacob Alfa, Ngabea Murtala, Bolaji Abdullahi, Ada Nwankwo, Anita Nnamonu

Methodology: Dike Ojji, Elena Libhaber, Karen Sliwa

Supervision: Karen Sliwa, Elena Libhaber

Validation: Karen Sliwa, Elena Libhaber

Writing – review and editing: Dike Ojji, Karen Sliwa, Elena Libhaber, Jacob Alfa, Ngabea Murtala, Bolaji Abdullahi, Ada Nwankwo, Anita Nnamonu

Writing – original draft preparation: Dike Ojji, Karen Sliwa, Elena Libhaber

## SOURCES OF FUNDING

Dr Dike Ojji was supported for a postdoctoral PhD Scholarship from Non‐Communicable Disease (NIH USA) TW008330‐01A1.

## CONFLICT OF INTEREST

None declared.
